# The Aura-Gain laryngeal mask for airway management in neonatal inguinal hernia surgery. A feasibility study

**DOI:** 10.48101/ujms.v128.9234

**Published:** 2023-02-27

**Authors:** Ali-Reza Modiri, Robert Frithiof, Tomas Luther, Peter Frykholm

**Affiliations:** Department of Surgical Sciences, Section of Anaesthesiology and Intensive Care Medicine, Uppsala University, Uppsala, Sweden

## Background

The benefits of the laryngeal mask airway (LMA) for elective procedures requiring general anesthesia in children are well documented. Besides easy insertion, the incidence of perioperative respiratory adverse events has been found to be lower when the LMA is compared to endotracheal intubation (1). However, there is a paucity of studies in small infants and neonates. In our tertiary pediatric anesthesia center, LMA is the most commonly used airway adjunct. When a new second generation LMA became available, we decided to perform a feasibility study of using the Aura-Gain LMA in infants undergoing inguinal hernia surgery.

## Aim

To investigate the feasibility and safety of using the Aura-Gain LMA in infants undergoing inguinal hernia surgery.

## Methods

The design was prospective observational. The regional ethics committee granted permission (dnr 2019-04418); informed consent was obtained from both parents. Neonates of post menstrual age ≤ 60 weeks scheduled for inguinal surgery were eligible for inclusion. Exclusion criteria were moderate or severe respiratory disease or congenital heart disease requiring treatment. Primary outcome was the successful completion of surgery. Secondary outcomes were time to correct placement, fiberoptic view of the larynx through the LMA, leak pressure, and adverse events associated with airway management. For the secondary outcomes, we started the clock when a decision to insert the LMA was made by the attending anesthesiologist and stopped it when an endtidal CO_2_ trace of a complete breath was visualized on the anesthesia monitor. We recorded visualization of the larynx using a fiberoptic endoscope inserted past the major bend of the AuraGain, using the Cormack-Lehane scale adapted for flexible laryngoscopy through the LMA, and we measured the leak pressure by increasing the peak pressure in steps of two up to a maximum of 30 cmH_2_0. The leak pressure was defined as the pressure when the difference between the inspired and expired tidal volumes was more than 10%.

The anesthesia technique included i.v. or mask induction according to the availability of easy i.v. access, maintenance with sevoflurane in oxygen targeting FiO_2_ 0.3, flow 0.2–1 L min^-1^. An 8 French feeding tube was inserted through the side-port of the LMA, initially used for suctioning the stomach and then left open during the procedure. Near the end of the procedure, sevoflurane was turned off, flows increased and bolus doses of propofol 0.5 mg kg^-1^ were given until the end-tidal sevoflurane concentration was ≤0.3, at which point the LMA was removed.

## Results

We included 20 neonates, with mean gestational age 50.2 ± 4.9 weeks, body weight 5.6 ± 1.0 kg (range 4.3–7.7) (Table 1). All operations were performed according to plan. All LMAs were inserted at the first attempt. Mean time to correct position from decision to intubate was 14.1 ± 6.3 s. We recorded a modified Cormack Lehane grade 1 in 17 infants and grade 2 in three infants respectively. There was no case of airway obstruction due to malpositioning or folding of the epiglottis. There were neither any adverse events at induction nor during LMA insertion. Pressure support ventilation was used initially in all infants, but in three cases a small dose of atracurium was given on request from the surgeon due to difficult operating conditions during pneumoperitoneum. There was one case of transient stridor after extubation, resolving spontaneously after a brief period of observation in the operating room.

**Table 1:** Patient/procedure characteristics and observations using the Aura-Gain laryngeal mask airway (Mean ± SD or absolute number).

Gestational age (weeks)	50.3 ± 4.9
Weight (kg)	5.6 ± 1.0
Duration of anesthesia (min)	96 ± 18
Duration of surgery (min)	34 ± 14
Laparoscopic/open hernioraphy Insertion time (seconds)	14.1 ± 6.3
Modified Cormack-Lehane grade 1 vs 2	17 vs 3
Oropharyngeal leak pressure (cmH2O)	16.8 ± 4.8
Time to first feeding in PACU	63 ± 49

PACU; Post Anesthesia Care Unit.

## Discussion

This small observational study corroborates previous reports of low rates of adverse events when LMAs are used in children. Drake-Brockman et al. reported a rate of perioperative respiratory adverse events (PRAE) of 18% vs 55% in infants with LMA vs endotracheal tube (ETT), respectively, in a randomized controlled trial (RCT) in older children (1). In an observational trial comparing spinal anesthesia to general anesthesia with LMA, the authors reported one case of bradycardia and one case of laryngospasm in the GA cohort (2). In the present study, the age and size of the infants were lower or similar compared to the former studies (1, 2). Despite this we found an even lower rate of adverse events and observed no serious PRAEs such as laryngospasm or hypoxemia. Furthermore, we had no cases of mucosal damage or bleeding and the majority of children breast- or bottle-fed within an hour of extubation. All LMAs were inserted on the first attempt in contrast to two randomized controlled trials comparing the AuraGain to the LMA Supreme in slightly older children, in which first attempt success-rate for the AuraGain was 96 and 86% respectively (3, 4). In a randomized controlled study comparing the LMA Supreme with the ProSeal LMA in infants, the rate of mucosal hyperemia, mucosal damage or blood on the LMA was 5% vs 8.3% in the two respective groups (5). Furthermore, the mean oropharyngeal leak pressures (OLP) were 17.2 and 24.1 cmH_2_0 in the two abovementioned RCTs comparing the AuraGain to the LMA Supreme (3, 4). Lopez-Gil et al. found that four different methods for detecting OLP were well correlated (6). Therefore we could speculate that the slightly lower mean OLP of 16.8 cmH_2_0 found in the present study may be attributed to the lower age of the infants rather than the measurement method.

The main limitation of the present study is that the sample size is small. Furthermore, we did not compare the Aura-Gain to other LMAs or ETT, and the above discussion on adverse events must therefore be interpreted with caution. The strength of the study is that it was performed in a homogenous cohort of small infants <60 weeks post menstrual age, which is younger than other published studies.

## Conclusion

We conclude that the AuraGain may be used for neonatal hernia surgery, since we found it easy to insert to correct position and there were no serious adverse events.
